# FoxP3 isoforms and PD-1 expression by T regulatory cells in multiple sclerosis

**DOI:** 10.1038/s41598-018-21861-5

**Published:** 2018-02-27

**Authors:** Manolo Sambucci, Francesca Gargano, Veronica De Rosa, Marco De Bardi, Mario Picozza, Roberta Placido, Serena Ruggieri, Alessia Capone, Claudio Gasperini, Giuseppe Matarese, Luca Battistini, Giovanna Borsellino

**Affiliations:** 1 Laboratory of Neuroimmunology, Fondazione Santa Lucia, Rome, 00143 Italy; 2 Institute of Experimental Oncology and Endocrinology, National Research Council (IEOS-CNR), Treg Cell Lab, Naples, 80131 Italy; 3 Department of Neurosciences, San Camillo Forlanini Hospital, Rome, 00152 Italy; 4 Department of Neurology and Psychiatry, Sapienza University of Rome, Rome, 00189 Italy; 5 Laboratory of Neuroembryology, Fondazione Santa Lucia, Rome, 00143 Italy; 6 Department of Molecular Medicine and Biotechnologies, University of Naples “Federico II”, Naples, 80131 Italy

## Abstract

Forkhead box P3 (FoxP3)+ regulatory T cells (Treg) are powerful mediators of immune regulation and immune homeostasis. In humans, Tregs are a heterogeneous population expressing surface markers which define phenotypically and functionally distinct subsets. Moreover, it is now clear that intracellular staining for FoxP3 does not unequivocally identify “true” suppressor cells, since several FoxP3 isoforms exist, and different reagents for FoxP3 detection are available. Here, we propose a strategy to identify potentially functional and suppressive Treg cells in an autoimmune disease like multiple sclerosis, and we suggest that in patients affected by this disease these cells are both reduced in number and functionally exhausted.

## Introduction

At every moment since its appearance in the developing organism, the immune system is in constant activity, continuously adapting itself to the environment and responding to environmental cues which determine its “configuration” at any given moment. To balance these perpetual challenges and unceasing activating signals, regulatory mechanisms exist which control the extent of immune activation, shutting down immune responses once the threat has been eliminated^1^. T regulatory lymphocytes are a fundamental component of these control mechanisms, and they represent a population of suppressor cells that contain autoreactive and over-shooting inflammatory immune responses by active suppression. Several subsets of T regulatory lymphocytes have been identified in humans and in experimental animals; their common feature is the ability to inhibit the effects of immune activation, such as proliferation or cytokine production by effector cells of both the innate and the adaptive arms of the immune system. It is now clear that conventional lymphocytes may acquire regulatory functions following stimulation in the presence of the appropriate cytokine milieu. However, the thymus hosts the development of a distinct lineage of CD4^+^ lymphocytes naturally committed to suppressive functions: natural T regulatory cells (Treg)^2,3^.

The key transcription factor controlling T cell development and function is FoxP3, and its deficiency determines highly aggressive systemic autoimmunity, both in mice and in humans^4–6^. Contrary to murine Treg cells, however, human Tregs are not homogeneous in gene expression, phenotype, and suppressive functions^7^. Moreover, in humans several splicing variants of FoxP3 have been described^8–11^, adding to the heterogeneity of the human Treg landscape. Indeed, two main isoforms are expressed at equivalent levels by Treg cells: one is the full-length isoform (FoxP3fl), while the other lacks exon 2 (FoxP3Δ2), which contains the sequences involved in the interaction with retinoic acid-related orphan receptor α and γt (RORα and RORγt). The main functional distinction between these two isoforms consists in the inability of FoxPΔ2 to interact with RORα^12^ and RORγt^13^ and to inhibit their function, ultimately contrasting the development of Th17 cells. A third isoform has also been described which lacks both exon 2 and exon 7 (FoxP3Δ2Δ7), which contrary to the other two isoforms facilitates Th17 differentiation^14^. The factors that regulate the generation of alternatively spliced isoforms include metabolic determinants, such as the impairment of the glycolytic pathway with consequent accumulation of the glycolytic enzyme enolase 1 in the nucleus and its binding to the FOXP3 promoter^15^, and exposure of T cells to the proinflammatory cytokine IL1β^14^.

Several studies have revealed that quantitative or qualitative declines in Treg cells contribute to the development of autoimmune diseases, although given the vast heterogeneity and complexity of these disorders a consensus has not been reached, and conflicting results have often been generated^16^.

The precise identification of natural T regulatory cells in the peripheral blood is in itself a challenge, since proteins expressed by T regulatory cells are mostly shared by activated conventional effector cells. However, in *ex-vivo* freshly isolated lymphocytes, the expression of certain combinations of markers neatly pinpoints distinct subsets of Tregs with varying suppressive abilities. Following the first characterization of human Tregs^17^, several studies have identified markers which are predominantly expressed – or selectively downregulated - by these cells^18–23^.

Miyara and colleagues^8^ have shown that CD45RA is a useful marker when combined with CD25 and FoxP3 expression to study the heterogeneity of Treg cells. In particular CD4^+^ CD45RA^−^CD25^hi^ cells show potent suppressive activity and the highest levels of  FoxP3 expression.

Previous observations by our lab^22^ have shown that the catalytic inactivation and conversion of extracellular ATP by CD39 is an anti-inflammatory key mechanism of Treg cells with implications in immune suppression, and that coexpression of CD39, CD45RO, and CCR6 identifies a confined subset of activated effector/memory-like suppressor cells^24^.

Based on recent data on the functional consequences of the differential expression of the distinct FoxP3 isoforms, and thanks to the availability of isoform-specific antibodies, we have investigated FoxP3 expression by Treg cells in patients with multiple sclerosis (MS) and in healthy donors (HD), focusing on the Treg subtypes identified by differential expression of surface markers. Also, we have measured expression of the inhibitory receptor PD-1 by Treg subsets, adding another piece to the complex puzzle of the factors regulating Treg activity. Our data shows that both naïve and memory Treg cells, defined by the expression of surface markers, are reduced in frequency in MS patients. Moreover, in patients Treg cells mainly express the FoxP3 isoform lacking exon 2; additionally, these cells present high membrane levels of inhibitory PD-1.

## Results

### Identification of FoxP3^+^ cells using different antibody clones unveils differences in Treg frequencies

To analyze whether immune dysregulation in patients with MS is associated with a decline in Treg frequencies in the peripheral blood, we investigated the expression of FoxP3 by flow cytometry both in healthy donors (HD) and in MS patients. We detected FoxP3 using the commercially available antibody clones PCH101, 150D, 259D/C7, 236A/E7. The PCH101 antibody reacts with an epitope located at the amino terminus of human FoxP3, detecting all FoxP3 isoforms, while the 150D antibody recognizes only the epitope encoded by exon 2 (FoxP3-E2), thus identifying Tregs with immune-suppressive abilities; antibodies 259D/C7 and 236A/E7 recognize an epitope located downstream of exon 2^25^. Freshly isolated PBMCs were stained with these 4 antibody clones in conjunction with surface staining of CD4 and CD25. In order to minimize inter-experimental variation, staining conditions were kept strictly homogeneous and the flow cytometer was calibrated prior to each measurement. These conditions allow us to measure with confidence also the Median Fluorescence Intensity (MFI) for FoxP3 staining.

**Figure 1:**
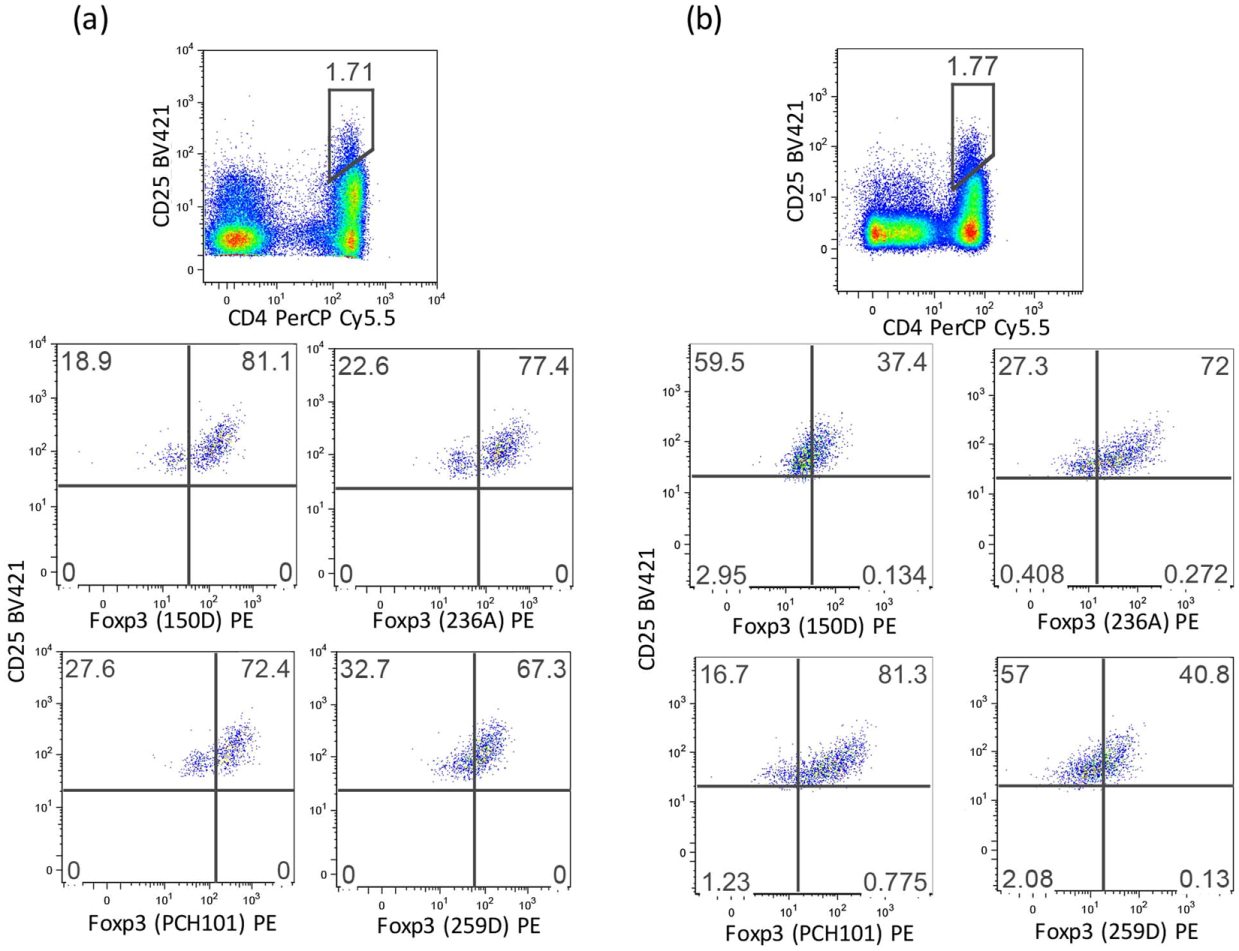
Representative plots showing the frequencies for Treg cells in PBMCs from a healthy donor (**a**) and an MS patient (**b**). PBMCs from the same sample were stained in 4 replicates with the different anti- FoxP3 clones (150D, 236A, 259D, PCH101). Numbers indicate percent of cells in each quadrant.

**Figure 2:**
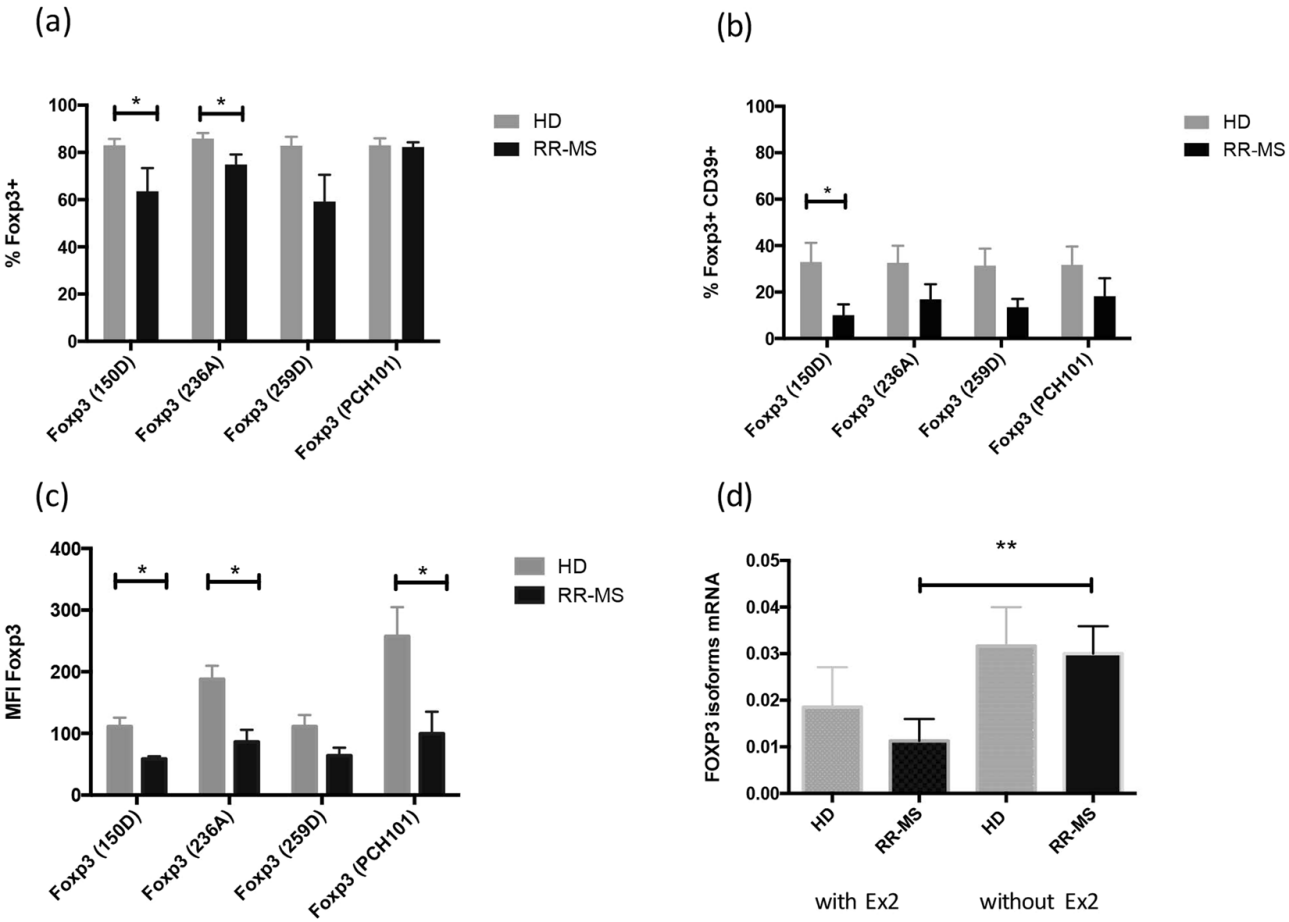
Frequencies and Median Fluorescence Intensity (MFI) of FoxP3^+^ cells within CD4^+^CD25^bright^ cells (**a**,**c**) or CD4^+^CD25^bright^CD39^+^ cells (**b**) from the PBMCs of HD (grey bar, n = 6) or MS individuals (black bar, n = 6). (**d**) rtPCR quantification of FOXP3 transcripts expressing exon 2 (with Ex2), without exon 2 (without Ex2) on CD4^+^CD25^high^ sorted cells obtained from RRMS patients (n = 4) or healthy donors (n = 4). Significant values: *p < 0.05; **p < 0.001. Statistical comparisons were performed by Student’s paired T test.

The results show that the percentages of FoxP3^+^ Treg cells identified by gating on the CD4^+^CD25^high^ cells are more variable in MS patients compared to HD (Fig. 1a vs b). Indeed, we observed that in MS patients, intensity of FoxP3^+^ cells, which correlates to the number of molecules per cell (Fig. 2c), was significantly lower than that of healthy individuals.

To confirm that PCH101 and 150D/E4 recognize different FoxP3 isoforms, we sorted CD4^+^CD25^high^CD127^neg^ cells and performed Western blotting using both antibodies to detect FoxP3 in the same sample (suppl. Figure 1). Indeed, the 150D/E4 antibody identifies one single band, which correspond to the isoforms containing exon 2, while the PCH101 antibody identifies two distinct bands, at 44 and 47KDa, since it recognizes an epitope located at the N-terminus of the protein and thus binds to all isoforms, which have different molecular weights.

Previous studies have shown that expression of CD39 defines the subset of Treg cells endowed with the most powerful suppressive abilities^22,26,27^. To correlate the expression of FoxP3 isoforms with immune function, we determined the positivity for FoxP3 with the available anti-FoxP3 clones in combination with surface staining of CD39 (Fig. 2c). Within CD4^+^CD25^high^ cells, the fraction of FoxP3^+^ CD39^+^ identified with clone 150D was significantly lower in MS patients compared to healthy donors; staining with the other antibody clones revealed lower frequencies of CD39^+^FoxP3^+^ cells in MS patients, although statistical significance was not reached. Thus, Tregs from MS patients express lower amounts of the FoxP3-E2 isoform which confers full suppressive abilities.

The highest percentages of Treg cells were detected by staining with PCH101 (Fig. 1), while the use of the 150D clone led to detection of a lower fraction of FoxP3^+^ cells. In HD, the percentage of FoxP3^+^ cells is less dependent on the antibody clone used, and consistently 70–80% of CD4^+^CD25^high^ cells express FoxP3, while in MS patients the fraction of FoxP3^+^ cells is generally lower. Cumulative data show that CD25^high^FoxP3^+^ cell frequencies in MS patients and in healthy donors are significantly different when clone 150D is used, while the same numbers of FoxP3^+^ cells are detected with clone PCH101 (Fig. 2a). Moreover, MS patients consistently show reduced levels of FoxP3 expression as measured by MFI.

Finally, to confirm our flow cytometric results, we asked whether MS individuals are carrying less FOXP3 transcripts with exon 2. Fresh PBMCs obtained from HD and MS individuals were stained with a mixture of fluorochrome-conjugated antibodies (CD4, CD25, CD127, CD39, FoxP3) the fraction of CD4^+^CD25^high^ and CD4^+^CD25^neg^ from each individual was sorted to purity. Sorted cells were then used to perform real-time PCR for FOXP3 isoforms (Fig. 2d). Results showed no differences in total FOXP3 expression between HD and MS in agreement with the flow cytometric data. However, in CD4^+^ CD25^high^ cells from in MS patients we measured lower levels of FOXP3 isoforms carrying exon 2, confirming the data obtained staining with the different FOXP3 antibody clones.

### The lower percentages of FoxP3^+^ Treg cells in MS patients correlate with reduced frequencies of memory Treg cells expressing the FoxP3-E2 isoform

**Figure 3:**
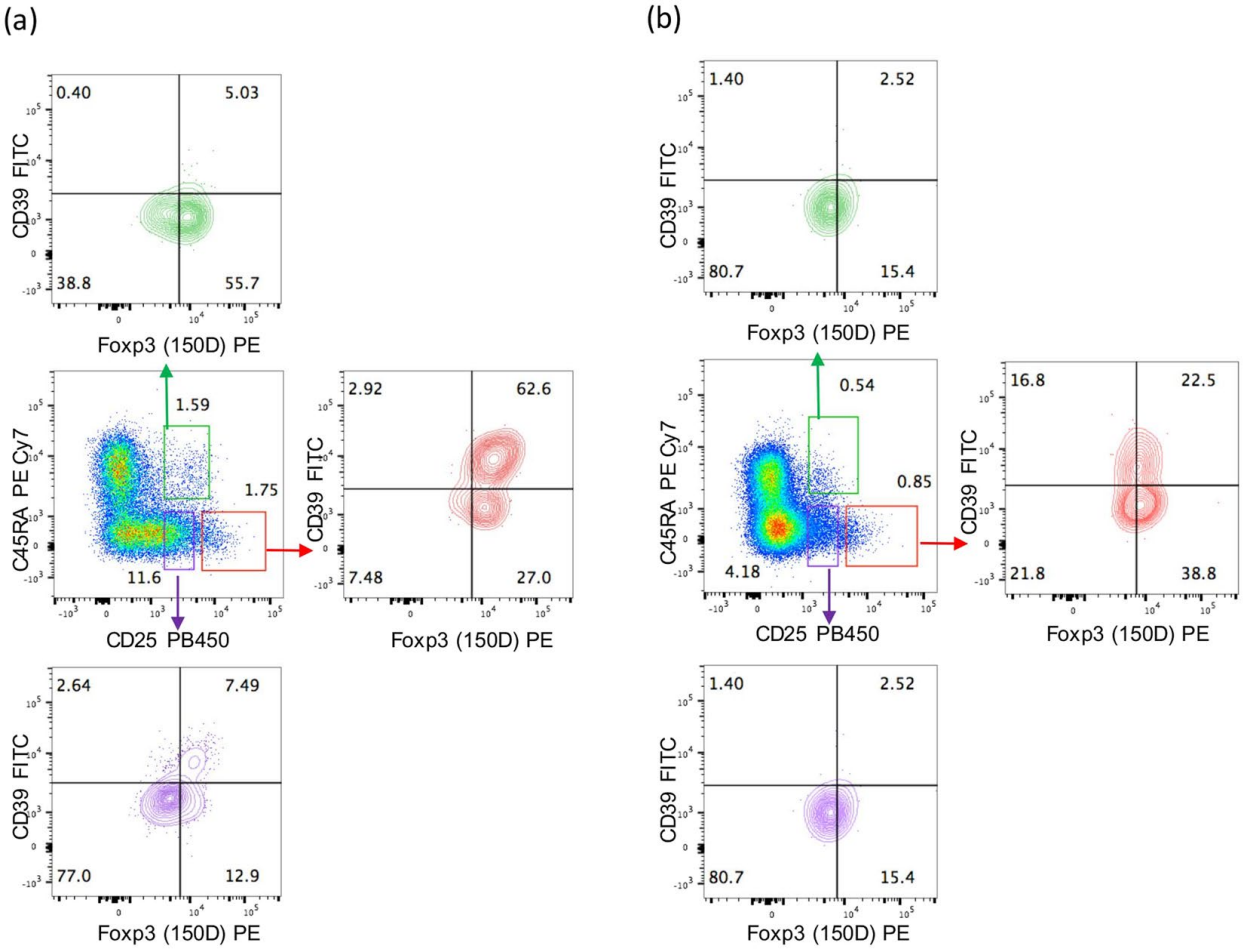
Definition of subpopulations of Treg cells by detection of cell surface molecules in one representative healthy donor (**a**) and one representative MS patient (**b**). PBMCs were stained for CD4, CD25, CD39, CD45RA, and FoxP3-exon2 (anti-FoxP3 clone 150D). Numbers indicate percent of cells in each quadrant. Three main subsets of CD4^+^ cells containing different levels of FoxP3 were defined by the expression of CD45RA and CD25: naïve Treg cells (CD45RA^+^ CD25^+^), memory Treg (CD45RA^−^ CD25^+^), activated Treg (CD45RA^−^ CD25^++^). In each subset the combination with CD39 and FoxP3-exon2 identifies true Treg cells.

**Figure 4:**
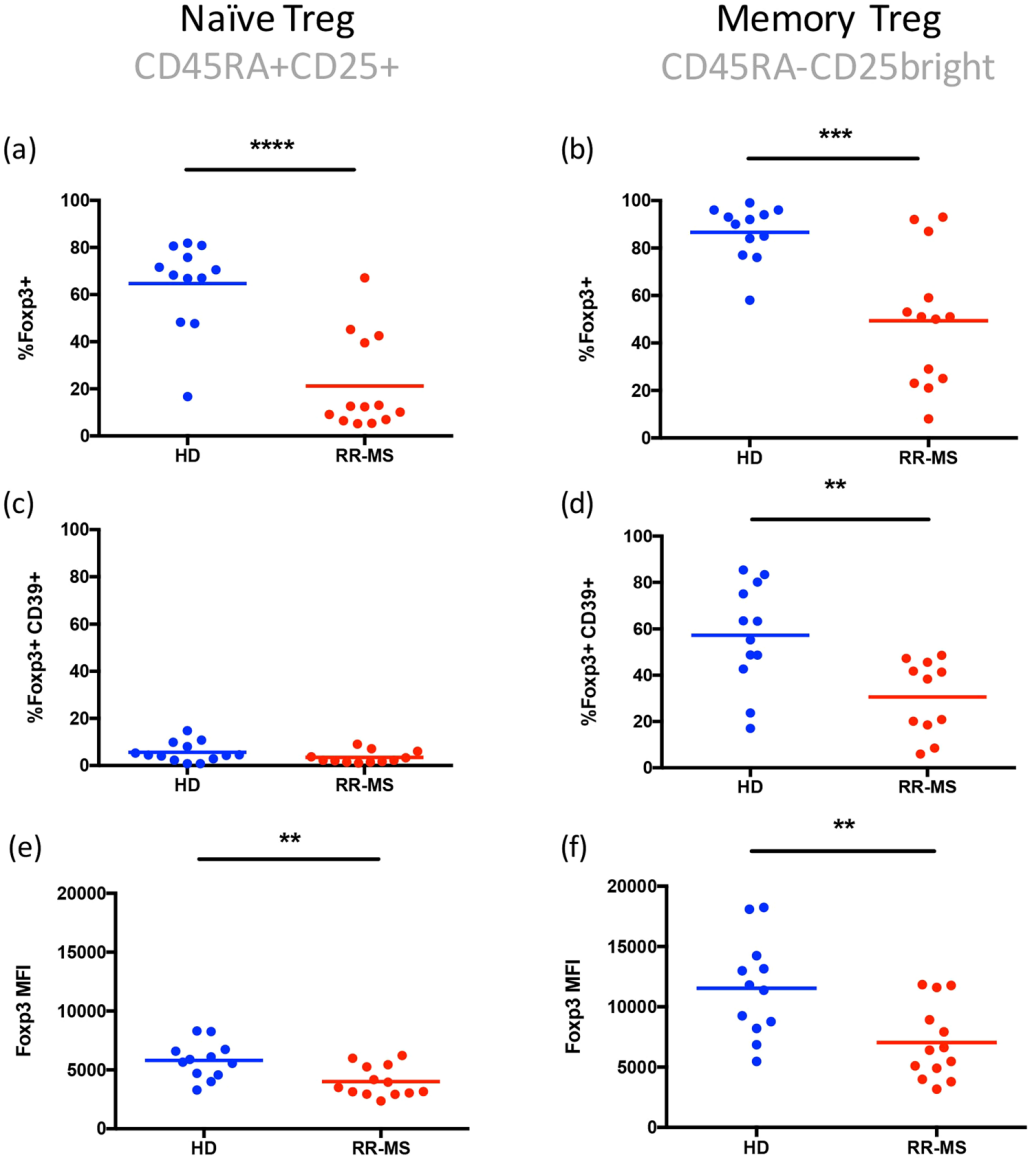
Percentage of FoxP3^+^ (**a**,**b**) and of FoxP3^+^CD39^+^ cells (**c**,**d**) within naïve and memory Treg cells. MFI of FoxP3 in naïve (**e**) and memory (**f**) Treg cells. Blue dots: healthy donors (n = 12); red dots: MS patients (n = 13). Statistical comparisons were performed by Student’s unpaired T test. Significant values: **p < 0.005, ***p < 0.001, ****p < 0.0001.

Given that expression of the FoxP3 splicing variant containing exon 2 has been associated with immune-suppressive abilities, and that the antibody clone 150D recognizes precisely an epitope encoded by a sequence present in exon 2, we performed a phenotypic study of Treg cells in PBMCs obtained from healthy individuals and MS patients, with the aim of clearly defining the cell subsets expressing FoxP3-E2 within CD4^+^CD25^high^ cells. Following the strategy for the identification of distinct Treg subsets proposed by Miyara and colleagues^8^, we stained cells with CD4, CD25, and CD45RA and defined three main subsets of cells within CD4^+^CD25^+^ lymphocytes: CD25^high^CD45RA^−^ memory Treg, CD25^+^CD45RA^+^ naïve Treg, and CD25^low^CD45RA^−^ activated T cells. Expression of CD39 and of the FoxP3-E2 isoform was then evaluated in each subset (Figs 3 and 4). The subset which was most enriched in FoxP3^+^ cells, as expected, was the CD25^high^CD45RA^−^ memory Treg, in both cohorts of individuals. However, MS patients showed a statistically significant reduction of these cells (Fig. 4b). Also, FoxP3 expression levels were higher in healthy individuals compared to MS patients. The analysis of CD39 expression confirmed that this marker is prevalently present on memory Treg cells (Fig. 4d), and more so in healthy individuals. Interestingly, also within the naïve Treg compartment FoxP3-E2 expression was significantly reduced in MS patients (Fig. 4a). As expected, CD39 expression was lowest in naïve Treg cells, in both HD and MS patients (Fig. 4c). Overall, these results indicate that the antibody clone 150D, used in combination with surface markers that define Treg subsets, reveals a reduction of distinct subsets of Treg cells in MS patients.

### The deficiency in memory Treg exon-2 in MS is associated with highest expression of PD-1

**Figure 5:**
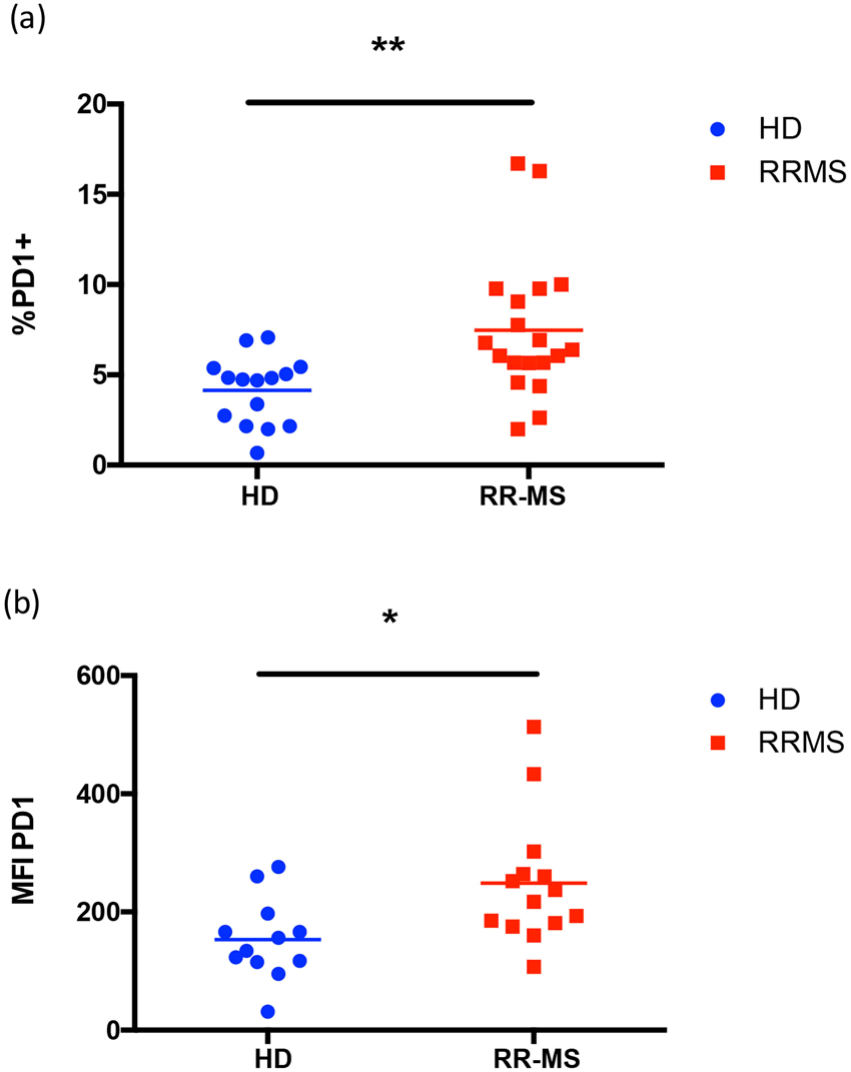
PD-1 expression measured as percent of positive cells (**a**) or MFI (**b**). PD-1 expression was measured on cells gated on blue dots: healthy donors (n = 12); red dots: MS patients (n = 13). Statistical comparisons were performed by Student’s unpaired T test. Cells were gated on CD4^+^CD25^bright^FoxP3-exon2^+^ *p < 0.05, **p < 0.005.

CD4^+^CD25^bright^FoxP3^+^ Treg cells, similar to conventional CD4^+^ T cells, express specific coinhibitory and costimulatory receptors involved in signaling pathways that modulate their functions. One such receptor is programmed cell death protein 1 (PD-1), which is expressed upon T cell activation, and provides a negative feedback to the effector functions of T cells during inflammation. It was recently demonstrated^28^ that human circulating Treg cells expressing high amounts of PD-1 are impaired in their ability to suppress CD4^+^ effector T cells. Indeed, high PD-1 expression identifies a population of dysfunctional, IFNγ-producing Treg cells, which are present also in healthy individuals, and which have been found to be expanded in tumor infiltrating Treg cells in malignant gliomas^28^. Thus, to further characterize the features of circulating Treg in MS individuals, we evaluated the expression of PD-1 in CD4^+^CD25^bright^CD45RA^−^ cells by flow cytometry, in PBMCs obtained from MS and HD. As shown, this subset comprises the majority of FoxP3^+^CD39^+^ effector Treg cells. Results show that Treg cells from MS patients express significantly higher levels of PD-1 compared to HDs (Fig. 5a and b), denoting an exhausted and dysfunctional status. Expression of PD-1 by the other Treg subsets was comparable between patients and healthy controls (not shown).

## Discussion

Treg cells play a central role in the maintenance of immune homeostasis, and their immune-suppressive functions are driven by the expression of the master regulator gene FOXP3. The central role of the FoxP3 transcription factor in maintaining immune homeostasis is best exemplified by the systemic autoimmune disease IPEX syndrome which affects individuals carrying mutations in FOXP3^5^. Several isoforms of FoxP3 have been described, arising from alternative splicing. A large number of instances has uncovered the role of alternative splicing in shaping cellular responses and in contributing to defects in immune function^29^, and here we have performed a study to characterize the FoxP3 isoforms present in MS patients in correlation with Treg surface proteins as surrogate markers for immune and cellular function. Of the known Foxp3 isoforms, the FoxP3Δ2 isoform lacks exon 2, which contains sequences crucial for the interaction with two transcription factors, RORα and RORγt, who have been shown to regulate differentiation of pro-inflammatory Th17 cells^12,13,30^. Th17 cells have been involved in the pathogenesis of several autoimmune disorders, as MS^31–33^. Interestingly, the close developmental interrelationship between immune-suppressive Tregs and proinflammatory Th17 cells allows shifts between a regulatory and an inflammatory condition, depending on environmental cues^13,34,35^. Defects in the Treg cell population have been shown to underlie several autoimmune disorders, although conflicting results concerning the nature and the quality of this deficiency have been reported in the literature^16^. More recently, also, several reports have assigned a negative prognostic value to tumor-infiltrating Treg cells, although in some tumor sites the presence of Treg cells is associated with improved survival^36^. These conflicting results may be due to variability in the detection of Treg cells with different reagents and read-outs, hindering the proper understanding of the mechanisms controlling immune regulation in different settings.

Here, by using stringent methods for the identification of human Treg cells, we show that in patients affected by multiple sclerosis there exists a significant reduction of Treg cells expressing the FoxP3-E2 isoform. Moreover, we show that in MS patients there is a reduction of both naïve and memory Treg cells, with memory Tregs displaying an increased expression of PD-1, a cell-exhaustion marker.

We find that in MS patients a lower fraction of Treg cells expresses the FoxP3-E2 isoform, compared to healthy donors, and this may deprive Treg cells of a further level of immunoregulation, through the inability to inhibit the transcription factors RORα and RORγt and the consequent failure to prevent the development of proinflammatory cells. The lower frequency of FoxP3^+^ cells identified with the antibody clone specific for exon 2 in MS patients may thus explain the decreased immunoregulatory abilities of Treg cells in this disease^37–40^. A possible explanation for the events which lead to splicing of the full-length FoxP3 protein may be the metabolic imbalances present in patients with chronic inflammatory diseases, as previously suggested^15^. These results are in agreement with previous work reporting that a reduction of functionally effective Treg cells underlies the pathogenesis of autoimmunity^37–40^.

Furthermore, we find that Treg cells isolated from MS patients express high levels of PD-1, confirming the notion that in these individuals the immune system is chronically activated in waves (such as those which occur during a chronic viral infection) and thus regulatory T cells become functionally exhausted, and as the disease proceeds immunoregulation may become compromised also due to upregulation of inhibitor molecules such as PD-1. Treg cells expressing high levels of PD-1 have been described in tumor settings^41^ and also in the context of autoimmunity^42^. Recently, stimulation through solely CD28 has been associated with high production of proinflammatory cytokines in MS patients^43^. Interestingly, CD28 has also been shown to be a crucial mediator of PD-1-induced inhibition of T cell function^44^. We have found a higher expression of PD-1 on regulatory T cells associated with a lower frequency of these cells in MS patients. Thus, in MS patients, probably due to chronic stimulation by unknown environmental agents (viral infections, dysbiotic gut flora), the subset of regulatory T cells is less represented in the peripheral blood than in healthy donors, and those few Treg cell which are still circulating are inhibited in their functions by the expression of PD-1.

In conclusion, this study underlines the importance of studying T regulatory cells taking in consideration the existence of distinct FoxP3 isoforms which identify cells with potentially diverse suppressive abilities and which may be differently expressed in health and in disease. The suppressive abilities of Treg cells are also influenced by the regulator of cell exhaustion PD-1, and the observation that Treg cells in MS patients express high levels of this molecule confirms previous findings of Treg dysfunction in autoimmunity.

## Methods

### Human samples and clinical specimens

Blood samples were collected from 13 MS patients with the relapsing-remitting form of MS (RR-MS), into 10 mL sodium heparin Vacutainer tubes (BD Biosciences, San Jose, CA) at the Neurology Department of the San Camillo Hospital, Rome, according to the guidelines and recommendations of the institution, which approved the study (6 male, 7 females, average age 43,6 range 32–55; EDSS 0–3,5). At the time of sampling, patients fell in the NEDA (no evidence of disease activity) category, following evaluation of clinical and neuroradiological parameters^45^. Healthy donors were recruited at the Fondazione Santa Lucia as approved by the Ethical Committee of the Institute (6 female, 6 male, average age 39.9, range 27–53). All patients and healthy donors provided informed consent.

PBMCs from HD and RR-MS were isolated by Ficoll and immediately stained for flow cytometric analysis or used as total cell lysates in Western Blot.

### Flow Cytometry of FoxP3 antibody clones staining and Treg phenotype

Fresh PBMC (*ex-vivo*) from MS patients and healthy controls were stained with CD4 PerCP-Cy5.5 (Biolegend), CD25 BV421 (Biolegend), CD39 FITC (Miltenyi Biotech), followed by fixation, permeabilization and intracellular staining with FoxP3 PE (clone 236A/E7, eBioscience; clone 150D/E4, eBioscience; clone 259D/C7, Becton Dickinson; clone PCH101, eBioscience). Live/Dead Fixable Aqua Dead cell stain (Invitrogen) was added to the cocktail of surface mAbs. Cells were acquired on a Beckman Coulter CyAn flow cytometer and analyzed using FlowJo 9.9.5 software. For the experiments on the Treg phenotype fresh PBMCs from MS and HD donors were stained with CD4APC-e780 (eBioscience), CD25 BV421 (Biolegend), CD45RA PE-Cy7 (Beckman Coulter), CD39 FITC (Miltenyi Biotech), FoxP3 PE (150D/E4, eBioscience), PD-1 BV650 (Becton Dickinson), CD127 APC-Alexa647 (Miltenyi Biotech), Live/dead cell stain. Cells were acquired on a Beckman Coulter CytoFLEX, and analyzed using Flowjo 10. Statistical analysis was performed using unpaired Student’s T test.

### Cell Sorting

PBMCs were isolated by Ficoll from healthy donor blood. Cells were then stained with CD4, CD25, and CD127 to identify Treg cells. Cells were sorted using a MoFlo cell sorter (Beckman Coulter). Purity of sorted cells was consistently >98%.

### Immunoblotting (Western Blot)

Total cell lysates were prepared in modified RIPA buffer (50 mM Tris/HCl, pH 8, 150 mM NaCl, 1% Nonidet P40, 0.5% sodium deoxycholate and 0.03% SDS) with 1 mM Na_3_VO_4_ and a cocktail of antiproteases from Sigma. A total 30–40 μg of proteins was separated by 8% SDS/PAGE and analyzed by Western blots. Membranes were cut vertically and incubated with different FoxP3 antibodies (PCH101 and 150D/E4, eBioscience). Immunoblots were analyzed by ECL with the ECL Western Blotting Reagent (Pierce). Tubulin α/β (Cell Signaling) was used as loading control.

### RNA isolation, RT-PCR and q-PCR

Total RNA was isolated from T cells with ReliaPrep™ RNA Cell - Miniprep System (Promega) following manufacture’s instructions, and 100 ng of cDNA was retro-transcribed using SuperScript™ II Reverse Transcriptase (Invitrogen).

The quantitative PCR was performed using the LightCycler^®^ 480 SYBR Green I Master Mix (Roche) and using specific couples of primers (Total FOXP3 FW: 5′-CAGCCATGATCAGCCTCACA-3′; Total FOXP3 REV: 5′-GCACTGGGATTTGGGAAGGT-3′; FOXP3 with Ex2 FW: 5′-CAGCTGCAGCTGCCCACACTG-3′; FOXP3 with Ex2 REV: 5′-GCCTTGAGGGAGAAGACC-3′; FOXP3 w/o Ex2 FW: 5′-CAGCTGCAGCTCTCAACGGTG-3′; FOXP3 w/o Ex2 REV:5′-GCCTTGAGGGAGAAGACC-3).

The amplification protocols of each couple of primers were tested before the analysis, shown below, and their specificity was confirmed in all assays by single peak performances of PCR products in melt curve analysis.

The amplification protocol for the total FOXP3 expression was 5 min 95 °C, (10 sec. 95 °C, 20 sec. 60 °C, 10 sec. 72 °C) × 45, and the amplification protocol for the FOXP3 with Ex2 and FOXP3 w/o Ex2 expressions was 5 min 95 °C, (10 sec. 95 °C, 20 sec. 56 °C, 10 sec. 72 °C). The gene expression was normalized to the expression of GADPH.

## Electronic supplementary material

Supplementary information
