# Supplementary material for: FoxP3 isoforms and PD-1 expression by T regulatory cells in multiple sclerosis
Source: Sci Rep. 2018 Feb 27;8:3674. doi: 10.1038/s41598-018-21861-5 (PMC5829149; doi:10.1038/s41598-018-21861-5)
Supplement: Supplementary file 1 — Supplementary information [file 41598_2018_21861_MOESM1_ESM.doc]

FoxP3 isoforms and PD-1 expression by T regulatory cells in multiple sclerosis.

Manolo Sambucci^1,2+^, Francesca Gargano^1+^, Veronica De Rosa^2^, Marco De Bardi^1^, Mario Picozza^1^, Roberta Placido^1^, Serena Ruggieri^3,4^, Alessia Capone^1,5^, Claudio Gasperini^3^, Giuseppe Matarese^2,6^, Luca Battistini^1*^, Giovanna Borsellino^1^

^1^ Laboratory of Neuroimmunology, Fondazione Santa Lucia, Rome, 00143, Italy.

^2^ Institute of Experimental Oncology and Endocrinology, National Research Council (IEOS-CNR), Treg Cell Lab, Naples, 80131, Italy.

^3^ Department of Neurosciences, San Camillo Forlanini Hospital, Rome, 00152, Italy.

^4^ Department of Neurology and Psychiatry, Sapienza University of Rome, Rome, 00189, Italy

^5^ Laboratory of Neuroembryology, Fondazione Santa Lucia, Rome 00143, Italy

^6^ Department of Molecular Medicine and Biotechnologies, University of Naples “Federico II”, Naples, 80131 Italy.

^+^ these authors contributed equally to this work

*corresponding author: l.battistini@hsantalucia.it

**Supplementary Fig.1** Immunoblotting with Foxp3 (**Left**, clone PCH101, eBioscience 14-4776-82, rat-anti-human FoxP3 recognizing all Foxp3 splicing isoforms, and **Right**clone 150D/E4 eBioscience 14-4774-82, mouse-anti-human-FoxP3, specific for the Foxp3-exon 2 isoform) showing the bands identified by the two clones, with their respective loading control (Tubulin, Rabbit-anti-human, Cell Signaling). The samples were derived from the same total cell lysates (CD4+CD25hiCD127neg T lymphocytes from healthy donors). After electrophoresis, samples were transferred on the same nitrocellulose membrane, which was cutted (grey dotted lane) to allow the incubation with the respective FoxP3 antibodies. Then filters were developed in the same film paying attention to re-align the molecular weight markers, with the same exposure timing (ECL, Thermo Scientific). One representative experiment of three independent is shown, normalized on total tubulin. Molecular sizes in KDa are indicated on the left in each blot. Original uncropped Western Blots are provided as additional material for Supplementary Fig.1

**Supplementary Fig.2** Schematic overview of Foxp3 exons and the spliced isoforms. It is showed antibody clones and binding sites for primers used for detection of each splice variant depicted.

Additional Material for Supplementary Fig.1

**Original Western Blots (uncropped) for Supplementary Fig.1**. Immunoblotting with FoxP3 (clone PCH101, eBioscience 14-4776-82 recognizing all FoxP3 splicing isoforms, or clone 150D/E4 eBioscience 14-4774-82 specific for the FoxP3-exon 2 isoform) showing the bands identified by the two clones, with their respective loading control (tubulin). One representative experiment of three independent is shown normalized on total tubulin. Lysates were derived from freshly isolated CD4+CD25brightCD127neg cells from the same sample. Molecular sizes in KDa are indicated on the left in each blot
